# Admission hyperglycemia, estimated glomerular filtration rate and outcome of acute ischemic stroke after mechanical thrombectomy: a mediation analysis

**DOI:** 10.3389/fneur.2026.1662158

**Published:** 2026-06-17

**Authors:** Dandan Geng, Yuntao Liu, Xueqian Xu, Linan Qiu, Jiahao Chen, Jincai He, Yisi Lin

**Affiliations:** 1 Department of Psychiatry, The First Affiliated Hospital of Chongqing Medical University, Chongqing, China; 2 Department of Neurology, The First Affiliated Hospital of Wenzhou Medical University, Wenzhou, Zhejiang, China; 3 Department of Neurology, The Third Affiliated Hospital of Wenzhou Medical University, Wenzhou, Zhejiang, China

**Keywords:** acute ischemic stroke, admission hyperglycemia, functional prognosis, mechanical thrombectomy, renal function

## Abstract

**Background and purpose:**

Multiple factors influence the efficacy of mechanical thrombectomy (MT) in patients with acute ischemic stroke. This study aimed to clarify the association between admission hyperglycemia (AH) and long-term functional outcome and to determine whether this association is mediated by postoperative renal function.

**Methods:**

A total of 397 patients with acute ischemic stroke who underwent MT between February 2018 and June 2024 and had available 6-month follow-up were included in this study. Functional outcome was assessed using the modified Rankin Scale (mRS), with scores of 0–2 indicating a good outcome. Multivariable regression models were constructed, adjusting for relevant covariates, including diabetes and the diabetes × AH interaction term. Mediation analysis was performed to explore the mediating effect of postoperative renal function.

**Results:**

Among the 397 patients, the median postoperative estimated glomerular filtration rate (post-eGFR) was 92.4 mL/min/1.73 m^2^ (IQR: 76.2–106.3), 198 (49.9%) patients had AH, and a good outcome at 6 months was achieved in 151 (38.0%) patients. After adjustment for confounding factors, multivariable regression analysis demonstrated that AH [OR (95% CI): 3.148 (1.672–5.925), *p* < 0.001] and lower post-eGFR [OR (95% CI): 0.978 (0.963–0.993), *p* = 0.004] were significantly associated with a lower likelihood of a good outcome at 6 months. Mediation analysis showed that post-eGFR mediated the association between AH and good prognosis, accounting for 25.57% of the total effect.

**Conclusion:**

Our study suggests that admission hyperglycemia is independently associated with long-term functional outcome after MT in patients with acute ischemic stroke, with or without diabetes. This association may be partly mediated by postoperative renal function.

## Introduction

Acute ischemic stroke (AIS) is a common cerebrovascular disease, with high morbidity, mortality, and disability rates, imposing a substantial economic burden to society and families (1). Studies show that every 6 s, someone in the world dies from a stroke (2). Mechanical thrombectomy (MT), a widely used standard endovascular treatment (EVT) for acute ischemic stroke, has substantially improved patient outcomes by enabling treatment in selected patients up to 24 h after symptom onset in selected patients (3). However, due to many factors affecting the treatment effect of stroke patients, not all patients achieve favorable long-term outcomes. In addition, long-term outcome data in AIS patients remain limited of AIS patients with relatively stable physical recovery level. Therefore, it is necessary to explore and understand the factors related to the long-term treatment outcome of MT to help clinical practice develop more targeted and effective treatment plans.

Abnormal blood glucose is common in AIS patients. Admission hyperglycemia (AH) may reflect both pre-existing diabetes mellitus and acute stress responses. However, it is reported that as many as two-thirds of nondiabetic patients receiving surgical treatment may also present with elevated admission blood glucose, which is a transient stress response against the onset of AIS, and may occur more in severe AIS patients with poor collateral circulation (4–6). Studies have shown that patients with abnormal blood glucose prior to EVT have worse postoperative outcomes (7–9). Hyperglycemia may adversely affect outcomes of AIS patients by changing blood–brain barrier permeability, damaging cerebrovascular reactivity, and increasing anti-fibrinolysis (10, 11). However, findings from the MR CLEAN trial (the Dutch Multicenter Randomized Clinical Trial of Endovascular Therapy for Acute Ischemic Stroke) showed no evidence of an effect of blood glucose on the efficacy of EVT (12), so the relationship between admission blood glucose and functional outcome in AIS patients may be influenced by other factors.

Renal damage is also frequent in AIS patients, which is commonly manifested by decreased estimated glomerular filtration rate (eGFR). Previous studies have shown that renal dysfunction during hospitalization, and is a significant predictor of functional outcome in AIS patients, is associated with longer hospital stay, increased morbidity and mortality (13–15). Moreover, most patients with AIS have many comorbidities, which may predispose them to renal dysfunction. Among them, studies have shown that AIS patients with diabetes are more likely to have renal impairment after MT (16–18). However, in AIS patients, whether AH is associated with postoperative renal dysfunction for renal function after MT, and whether renal function affects the relationship between AH and the long-term prognosis of stroke remains uncertain.

The aim of this study was to clarify the association of AH, postoperative renal function, and long-term prognosis. We further sought to explore whether glycemic status may influence long-term outcomes by optimizing glycemic management in AIS. Therefore, our research we hypothesized that AH would be independently associated to long-term prognosis of MT patients, and the relationship may be mediated by postoperative renal function.

## Methods

### Study population

This prospective cohort study analyzed consecutive patients with acute ischemic stroke who underwent mechanical thrombectomy at the First Affiliated Hospital of Wenzhou Medical University from February 2018 to June 2024. During this period, patients were included if they met the following criteria: aged ≥18 years; diagnosis of AIS confirmed by CT or MRI; treatment initiated within 24 h of symptom onset; and pre-stroke modified Rankin Scale (mRS) score ≤2. Patients with pre-stroke mRS ≥ 3 were excluded to minimize confounding from pre-existing disability and to ensure that post-stroke functional outcomes would primarily reflect stroke-related injury.

### Data collection

Clinical data were collected from the electronic medical record system including demographic data, serum creatinine at admission and within 72 h after surgery, contrast volume used for imaging diagnosis and MT, admission blood glucose, time from onset to reperfusion (OTR), atrial fibrillation (AF), hypertension. Stroke subtypes were further categorized into anterior and posterior circulation based on neuroimaging findings, and occlusion locations were recorded when available. Patients were considered to have diabetes if the HbA1c was ≥6.5% during hospitalization or taking antidiabetic medications before or at the time of stroke (19). AH was defined as admission blood glucose ≥7.8 mmol/L (4, 20). In addition, eGFR was calculated with the Chronic Kidney Disease Epidemiology Collaboration equation based on serum creatinine (21). Post-procedure eGFR was defined as the lowest eGFR value within 72 h after mechanical thrombectomy, calculated based on serum creatinine measurements. eGFR was primarily treated as a continuous variable. mTICI score 2b and 3 means successful reperfusion. mRS, National Institutes of Health Stroke Scale (NIHSS), and ASPECTS were evaluated by professional neurologists. Meanwhile, Functional outcome was dichotomized, with a score of 3–6 considered to be functionally dependent or dead and a score of 0–2 indicating a better prognosis. Long-term follow-up was assessed at 6 months, which was assessed via structured telephone interviews conducted by trained neurologists who were blinded to baseline glucose and renal function. The study protocol was approved by the Ethics Committee of the First Affiliated Hospital of Wenzhou Medical University (YS2018002) and conducted in accordance with the ethical guidelines of the declaration of Helsinki in 1975. Written informed consent was obtained from all participants prior to enrollment. All data were fully anonymized before analysis to protect patient privacy.

### Statistical analyses

The continuous variables of normal distribution were are presented as mean ± SD and compared using Student’s *t*-test. The variables of skewness distribution were shown as median (interquartile range, IQR) and analyzed using Mann–Whitney *U* tests. Chi-square test was applied to compare differences between groups for categorical variables.

The statistical procedures mainly included: First, the association between AH, postoperative renal function and functional outcome was tested. Secondly, the variables with *p* < 0.1 in the univariate analysis based on mRS grouping, together with diabetes and diabetes × AH, were used to establish a multivariate regression model. Finally, we investigated whether the postoperative renal function was a mediator.

Given that the outcome was dichotomous, the Sobel test was used (22), which includes three independent regression equations in the first step. In the first regression equation, logistic regression was performed between the dependent variable (good functional outcome) and the independent variable (AH). In the second regression equation, linear regression was performed with postoperative eGFR (post-eGFR) as the dependent variable and AH as the independent variable. Logistic regression of the dependent variable (good functional outcome) against the independent variable (AH) and the mediating variable (post-eGFR) was performed in the third regression equation. All regression equations were adjusted for confounding factors, including variables with *p* < 0.1 in the univariate analysis as well as diabetes, diabetes × AH, and clinically relevant variables were also included based on prior literature and biological plausibility. In the second step, the regression coefficients calculated in the first step should be converted to a unified scale by a standardized method. The final step used the R Mediation software package to calculate indirect effects and 95% confidence intervals. When Z mediation > |1.96|, the test is significant.

A two-sided *p* < 0.05 was considered statistically significant. All data analysis was calculated by IBM SPSS statistical software Version 26 for Windows and R software (Version 4.3.2).

## Results

### Patients baseline characteristics

**Figure 1:**
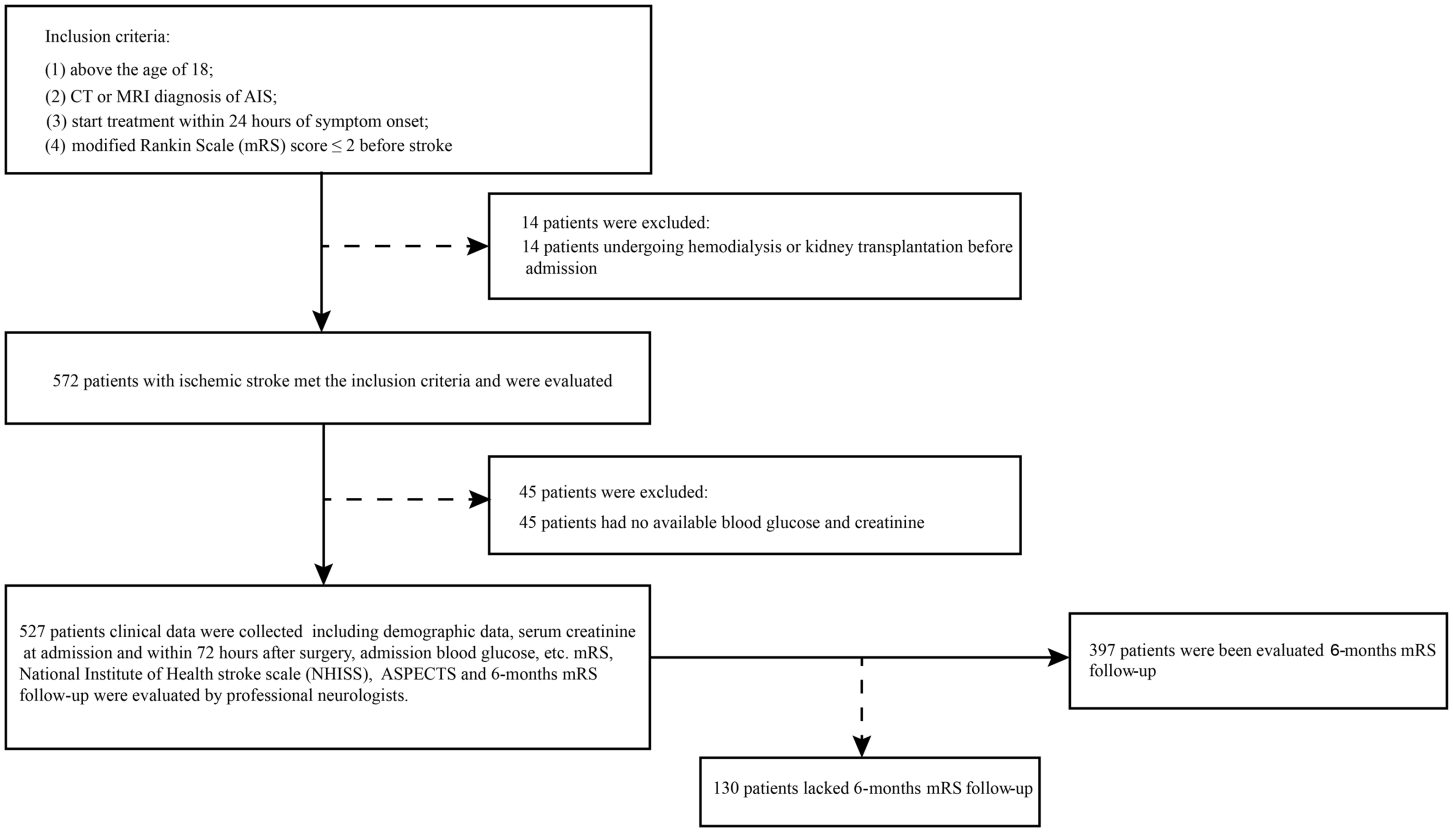
Flow diagram of patient selection. Flow diagram illustrating patient screening, inclusion, and exclusion. mRS, modified Rankin Scale; NIHSS, National Institute of Health stroke scale; ASPECTS, Alberta Stroke Program Early CT Score.

A total of 586 patients with AIS underwent MT between February 2018 and June 2024. A total of 586 patients were initially eligible for inclusion, 14 patients undergoing hemodialysis or kidney transplantation before admission were excluded, 45 patients had no available blood glucose and creatinine, and 130 patients lacked 6-month mRS follow-up data. Ultimately, 397 patients with available 6-month follow-up data were included in the final analysis. The loss to follow-up rate was 24.67% (Figure 1). Baseline characteristics of patients with good and poor outcomes are shown in Table 1. Among the 397 study patients, the mean age was 66.4 ± 12.3 years, 283 patients (71.3%) were male, the median post-eGFR was 92.4 mL/min/1.73 m^2^ (IQR: 76.2–106.3), 198 patients (49.9%) had AH. In addition, a good outcome at 6 months was achieved in 151 patients (38.0%) (Table 1).

**Table 1:** Baseline characteristics of patients treated with MT grouped by 6 month follow-up according to mRS.

Variables	Total (*n* = 397)	Good outcome (*n* = 151)	Poor outcome (*n* = 246)	*p-*value
Age, year (mean ± SD)	66.4 ± 12.3	62.2 ± 12.7	68.9 ± 11.4	<0.001^*^
Male, *n* (%)	283 (71.3%)	119 (78.8%)	164 (66.7%)	0.009^*^
OTR, h (median, IQR)	6.8 (5.3–8.7)	6.7 (5.3–8.2)	6.8 (5.3–9.1)	0.480
Smoking, *n* (%)	158 (40.2%)	72 (47.7%)	86 (35.5%)	0.017^*^
Drinking, *n* (%)	135 (34.4%)	58 (38.4%)	77 (31.8%)	0.181
Hypertension, *n* (%)	254 (64.3%)	90 (59.6%)	164 (67.2%)	0.125
Dyslipidemia, *n* (%)	130 (33.3%)	42 (28.4%)	88 (36.4%)	0.105
Diabetes, *n* (%)	101 (25.6%)	34 (22.5%)	67 (27.5%)	0.274
AH, *n* (%)	198 (49.9%)	50 (33.1%)	148 (60.2%)	<0.001^*^
AF, *n* (%)	110 (28.0%)	32 (21.3%)	78 (32.1%)	0.021^*^
Coronary disease, *n* (%)	32 (8.1%)	11 (7.3%)	21 (8.6%)	0.640
SBP, mmHg (median, IQR)	135.0 (120.0–153.8)	134.0 (121.0–151.0)	135.5 (118.0–156.3)	0.451
DBP, mmHg (median, IQR)	78.0 (68.0–90.0)	79.0 (69.0–90.0)	78.0 (68.0–90.0)	0.745
Pre-eGFR, mL/min/1.73 m^2^ (mean ± SD)	91.1 ± 20.3	93.2 ± 20.7	89.8 ± 20.0	0.111
Post-eGFR, mL/min/1.73 m^2^ (median, IQR)	92.4 (76.2–106.3)	99.1 (87.8–112.3)	87.4 (68.5–99.9)	<0.001^*^
Contrast, mL (median, IQR)	250 (200–300)	250 (200–300)	250 (200–300)	0.767
TOAST mechanism				0.492
LAA, *n* (%)	185 (46.6%)	76 (50.3%)	109 (44.3%)	
CE, *n* (%)	178 (44.8%)	61 (40.4%)	117 (47.6%)	
Others, *n* (%)	34 (8.5%)	14 (9.3%)	20 (8.1%)	
Baseline NIHSS, score (median, IQR)	16.0 (12.0–18.0)	15.0 (10.0–18.0)	16.0 (12.0–20.0)	0.019*
Baseline ASPECTS, score (mean ± SD)	8.0 ± 2.2	8.3 ± 2.3	7.9 ± 2.2	0.384
Successful reperfusion, *n* (%)	338 (92.6%)	131(95.6%)	207 (90.8%)	0.088

MT, mechanical thrombectomy; mRS, modified Rankin Scale; OTR, time from onset to reperfusion; AH, admission hyperglycemia; AF, atrial fibrillation; SBP, systolic blood pressure; DBP, diastolic blood pressure; pre-eGFR, pretreatment estimated glomerular filtration rate; post-eGFR, postoperative estimated glomerular filtration rate; TOAST, trial of ORG 10172 in acute stroke; LAA, Large artery atherosclerosis; CE, Cardioaortic embolism; Others, Other causes; NIHSS, National Institute of Health stroke scale.

^*^p values considered statistically significant; ^*^p < 0.05.

Several significant differences were observed between groups (Table 1). Participants with good outcome were more likely to be younger (mean ± SD: 62.2 ± 12.7 vs. 68.9 ± 11.4, *p* < 0.001) and had higher post-eGFR [median (IQR): 99.1 (87.8–112.3) vs. 87.4 (68.5–99.9) mL/min/1.73 m^2^, *p* < 0.001], a lower baseline NIHSS score [median (IQR): 15.0 (10.0–18.0) vs. 16.0 (12.0–20.0), *p* = 0.019], and had a lower prevalence of AF (21.3% vs. 32.1%, *p* = 0.021). In addition, patients with a good outcome were had a lower prevalence of AH than those with a poor outcome (33.1% vs. 60.2%, *p* < 0.001). However, OTR, drinking, hypertension, dyslipidemia, diabetes, pre-eGFR, contrast, and successful reperfusion did not differ significantly in Table 1.

### Baseline data of diabetic and nondiabetic patients stratified according to admission glucose

**Figure 2:**
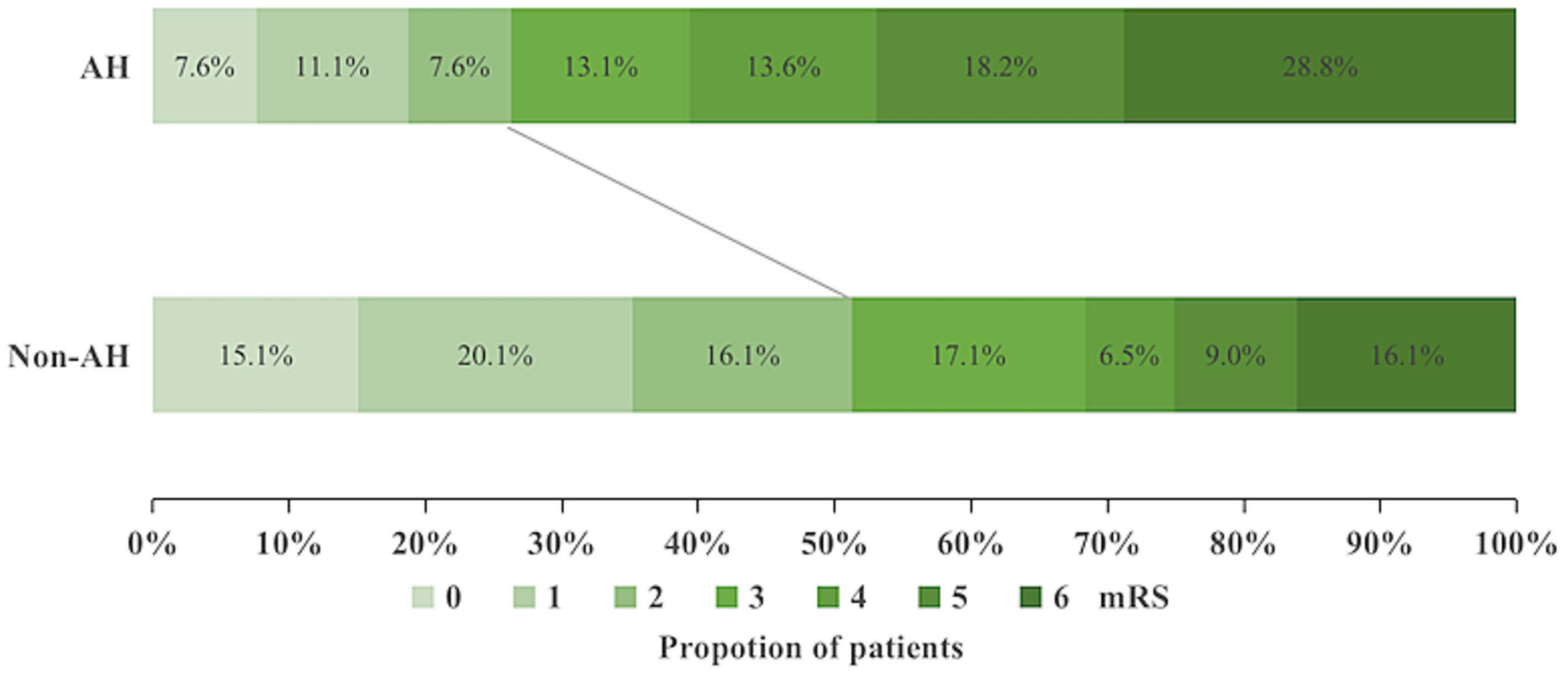
Association of admission hyperglycemia and functional improvement according to the distribution of mRS score at 6 months. According to the distribution of mRS scores, non-AH patients had better functional outcomes at 6 months (*p* < 0.001). AH, admission hyperglycemia; mRS, modified Rankin Scale.

**Figure 3:**
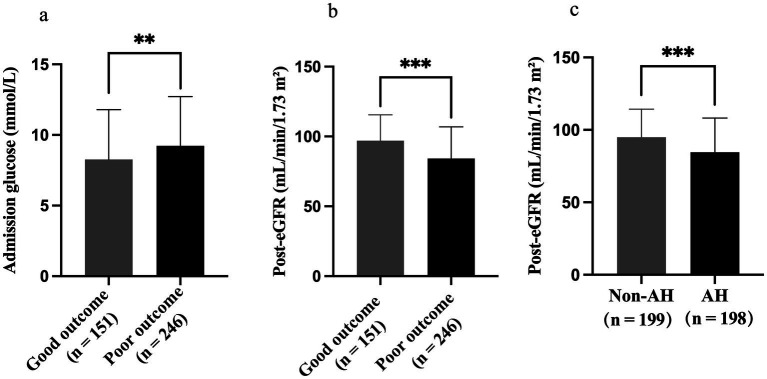
Association of admission glucose, post-eGFR, and functional outcome at 6 months. **(a)** AIS patients with improved functional prognosis tended to have lower admission blood glucose; **(b)** AIS patients with good outcome were more likely to have a higher post-eGFR; **(c)** compared with non-AH, the group of patients with AH had a lower post-eGFR. *p* < 0.005, *p* < 0.001.

The entire cohort was divided into nondiabetic (*n* = 295) and diabetic (*n* = 102) groups and further stratified into non-AH and AH subgroups (Table 2). The objective was to evaluate differences in baseline characteristics and clinical outcomes between AIS patients with and without diabetes mellitus according to admission glucose status. The results showed that AIS patients without AH tended to have higher post-eGFR (*p* < 0.001) and better functional outcomes (*p* < 0.001), regardless of diabetes status. Consistent with these findings, the distribution of mRS scores demonstrated that non-AH patients had better functional outcomes at 6 months (*p* < 0.001) (Figure 2). Among diabetic patients, those with AH were older (mean ± SD: 68.9 ± 10.6 vs. 52.1 ± 12.6, *p* = 0.001) and more frequently had dyslipidemia (71.6% vs. 28.6%, *p* = 0.001) and hypertension (77.3% vs. 42.9%, *p* = 0.006) than those without AH. In contrast, among nondiabetic patients, those with AH had higher baseline NIHSS scores compared with controls (Table 2). Figure 3 further illustrates the relationships among admission blood glucose, post-eGFR, and 6-month functional outcomes. Consistent with the above results, AIS patients with better functional prognosis had lower admission glucose levels and higher post-eGFR, whereas patients with AH exhibited lower post-eGFR (Figure 3).

**Table 2:** Baseline data of diabetic and nondiabetic patients stratified according to admission glucose.

Variables	Diabetic (*n* = 102)				Nondiabetic (*n* = 295)			
	Total	Non-AH (*n* = 14)	AH (*n* = 88)	*p*-value	Total	Non-AH (*n* = 185)	AH (*n* = 110)	*p*-value
Age, year (mean ± SD)	66.5 ± 12.3	52.1 ± 12.6	68.9 ± 10.6	<0.001*	66.3 ± 12.3	65.9 ± 12.3	66.9 ± 12.5	0.532
Male, *n* (%)	27 (26.5%)	1 (7.1%)	26 (29.5%)	0.074	208 (70.5%)	136 (73.5%)	72 (65.5%)	0.123
OTR, h (median, IQR)	6.8 (4.7–8.8)	8.1 (5.9–10.1)	6.4 (4.6–8.4)	0.050	6.8 (5.4–8.7)	6.8 (5.6–8.7)	6.6 (5.2–8.9)	0.558
Smoking, *n* (%)	40 (39.2%)	3 (21.4%)	37 (42.0%)	0.134	118 (40.0%)	81 (43.8%)	37 (33.6%)	0.082
Drinking, *n* (%)	27 (26.5%)	2(14.3%)	25(28.4%)	0.257	108 (36.6%)	68 (36.8%)	40 (36.4%)	0.937
Contrast, mL (median, IQR)	250.0 (200.0–300.0)	250.0 (187.5–350.0)	250.0 (200.0–350.0)	0.845	250.0 (200.0–300.0)	250.0 (200.0–300.0)	250.0 (200.0–300.0)	0.333
Dyslipidemia, *n* (%)	67 (65.7%)	4 (28.6%)	63 (71.6%)	0.001*	63 (21.4%)	34 (18.4%)	29 (26.4%)	0.112
AF, *n* (%)	29 (28.4%)	1(7.1%)	28 (31.8%)	0.055	81 (27.5%)	45 (24.3%)	36 (32.7%)	0.119
Coronary disease, *n* (%)	15 (14.7%)	1 (7.1%)	14 (15.9%)	0.382	17 (5.8%)	9 (4.9%)	8 (7.3%)	0.397
Hypertension, *n* (%)	74 (72.5%)	6 (42.9%)	68 (77.3%)	0.006*	180 (61.0%)	110 (59.5%)	70 (63.6%)	0.512
SBP, mmHg (median, IQR)	135.0 (117.5–152.5)	124.5 (110.5–136.8)	140.0 (120.0–156.0)	0.049*	135.0(120.0–154.0)	134.0 (119.3–153.8)	138.0 (120.0–156.0)	0.674
DBP, mmHg (median, IQR)	77.0 (68.5–88.0)	76.0 (71.0–88.3)	77.0 (68.0–88.0)	0.867	79.0 (68.0–90.3)	79.0(68.0–91.0)	79.0 (69.8–90.0)	0.646
Pre-eGFR, mL/min/1.73 m^2^ (mean ± SD)	92.9 ± 19.2	96.7 ± 22.6	92.3 ± 18.7	0.449	90.5 ± 20.7	91.4 ± 20.1	89.0 ± 21.6	0.334
Post-eGFR, mL/min/1.73 m^2^ (median, IQR)	93.6 (71.0–107.9)	119.6 (95.4–123.9)	89.4 (68.5–104.3)	0.001*	92.3 (77.2–104.8)	96.3 (81.5–107.1)	86.4 (68.2–100.3)	<0.001*
TOAST mechanism				0.132				0.195
LAA, *n* (%)	49 (48.0%)	9 (64.3%)	40 (45.5%)		136 (46.1%)	91 (49.2%)	45 (40.9%)	
CE, *n* (%)	45 (44.1%)	3 (21.4%)	42 (47.7%)		132 (44.7%)	80 (43.2%)	52 (47.3%)	
Others, *n* (%)	7 (6.8%)	2 (14.3%)	5 (5.7%)		26 (8.8%)	13 (7.0%)	13 (11.8%)	
Baseline NIHSS, score (median, IQR)	15.0 (12.0–18.0)	15.0 (12.5–20.5)	15.0 (12.0–18.0)	0.654	16.0 (12.0–18.0)	15.0 (11.0–18.0)	17.0 (13.0–19.0)	0.044*
Baseline ASPECTS, score (mean ± SD)	8.3 ± 2.2	7.7 ± 2.9	8.4 ± 2.1	0.286	8.0 ± 2.2	8.0 ± 2.2	7.9 ± 2.3	0.557
Successful reperfusion, *n* (%)	86 (84.3%)	10 (100%)	76 (86.4%)	0.419	250 (84.7%)	156 (84.3%)	94 (85.5%)	0.759
Hemorrhagic transformation, *n* (%)	51 (50.0%)	5(35.7%)	46(52.3%)	0.233	116 (39.3%)	66 (35.7%)	50 (45.5%)	0.104
Mortality (discharged), *n* (%)	28 (27.5%)	3 (21.4%)	25 (28.4%)	0.555	57 (19.3%)	35 (18.9%)	22 (20.0%)	0.784
mRS (6 months)				0.001*				<0.001*
0–2	34 (32.4%)	10 (71.4%)	24 (27.3%)		117 (39.7%)	91 (49.2%)	26 (23.6%)	
3–6	67 (65.7%)	4 (28.6%)	63 (71.6%)		177 (60.0%)	93 (50.3%)	84 (76.4%)	

AH, admission hyperglycemia; OTR, time from onset to reperfusion; AF, atrial fibrillation; SBP, systolic blood pressure; DBP, diastolic blood pressure; pre-eGFR, pretreatment estimated glomerular filtration rate; post-eGFR, postoperative estimated glomerular filtration rate; TOAST, trial of ORG 10172 in acute stroke; LAA, Large artery atherosclerosis; CE, Cardioaortic embolism; Others, Other causes; NIHSS, National Institute of Health stroke scale; mRS, modified Rankin Scale.

^*^p values considered statistically significant; ^*^p < 0.05.

### Associated factor of good outcome at 6 months

**Figure 4:**
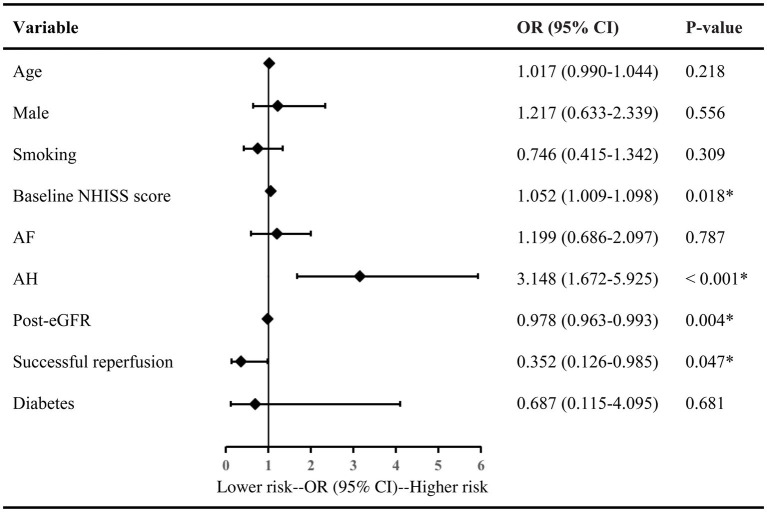
Associated factor of good outcome at 6 months. Multivariate logistic regression was adjusted for potential confounders, including variables with *p* values <0.1 in univariate analysis: age, sex, smoking, baseline NIHSS score, post-eGFR, successful reperfusion plus diabetes, diabetes × AH. NIHSS, National Institute of Health stroke scale; AH, admission hyperglycemia; AF, atrial fibrillation; post-eGFR, postoperative estimated glomerular filtration rate; OR, odds ratio; CI, confidence interval. *p values considered statistically significant; *p < 0.05.

In Figure 4, after multivariate adjustment for potential confounders, including variables with *p* values <0.1 in univariate analyses: age, sex, smoking, baseline NIHSS score, Post-eGFR, successful reperfusion as well as diabetes and the diabetes × AH interaction term, AH [OR (95% CI): 3.148 (1.672–5.925), *p* < 0.001] and lower post-eGFR [OR (95% CI): 0.978 (0.963–0.993), *p* = 0.004] were significantly associated with a decreased likelihood of good outcome at 6 months (Figure 4). In addition, baseline NIHSS score [OR (95% CI): 1.052 (1.009–1.098), *p* = 0.018] and successful reperfusion [OR (95% CI): 0.352 (0.126–0.985), *p* = 0.047] remained independently associated with good outcome.

### Mediation by post-eGFR of the association between AH and functional outcome

**Figure 5:**
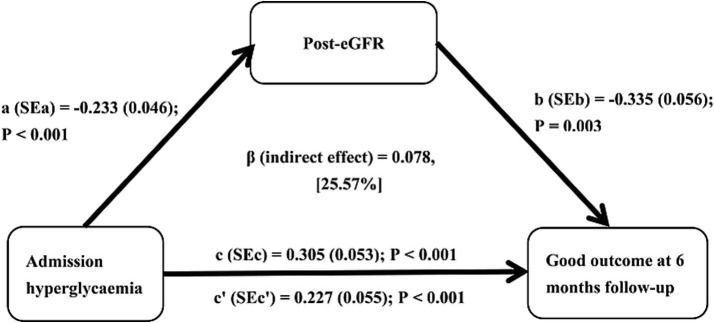
Mediation by post-eGFR of the association between admission hyperglycemia and functional outcome. The mediation model showed standardized path coefficients between admission hyperglycemia (AH), post-eGFR, and good outcome at 6 months; potential covariates (e.g., age, sex, smoking, baseline NIHSS score, atrial fibrillation, successful reperfusion, diabetes, diabetes × AH) were controlled. c’, regression coefficient of the path from admission hyperglycaemia to a good outcome at 6 months includes the post-eGFR adjusted. c, Regression coefficient of the path from admission hyperglycaemia to a good outcome at 6 months without adjustment for post-eGFR. All individual paths are satisfied.

To further explore the potential role of post-eGFR in the association between AH and functional outcome, a mediation analysis was performed. The results demonstrated that post-eGFR significantly mediated the association between AH and good outcome, with a mediating effect accounting for 25.57% of the total effect (Figure 5). In the multivariable analysis, poor long-term functional outcomes after MT in patients with AIS were significantly associated with AH, whereas a prior history of diabetes mellitus was not independently associated with outcomes. Furthermore, no significant interaction between diabetes status and AH was observed in the regression models, suggesting that the effect of elevated acute-phase glucose levels on prognosis was consistent regardless of pre-existing diabetes. These findings indicate that acute glycemic status at presentation, rather than chronic diabetic condition, may play a more critical role in determining long-term functional outcomes after MT (Figure 4).

## Discussion

To our knowledge, this study is the first to highlight the association between AH and 6-month functional outcomes after MT in patients with AIS. The results of this study suggest that AH is independently associated with long-term functional outcomes after MT in AIS patients, and this association is not modified by diabetes status, and this association is partially mediated by postoperative renal function.

Growing evidence indicates that poor glycemic control is an important factor associated with morbidity and mortality after vascular surgery (23). An observational study by Bruno et al. showed that hyperglycemia was associated with poor outcome after stroke (24). Our findings are consistent with prior reports by demonstrating that AH is associated with worse long-term functional outcomes after MT, regardless of the presence or absence of diabetes. We found that poor long-term functional outcomes after MT in AIS patients were associated with elevated acute-phase glucose levels rather than a prior history of diabetes mellitus, which was not an independent predictor after adjustment. Moreover, no significant interaction between diabetes and acute-phase hyperglycemia was observed, indicating that the adverse effect of hyperglycemia is consistent regardless of diabetes status.

These findings suggest that acute stress-related hyperglycemia may be more clinically relevant than chronic glycemic status in this context. Mechanistically, acute hyperglycemia may aggravate ischemic injury through oxidative stress, endothelial dysfunction, and blood–brain barrier disruption. The lack of an independent effect of diabetes and absence of interaction with hyperglycemia also imply no strong correlation between these variables in the acute stroke setting within our cohort.

However, the exact mechanisms by which AH contributes to poor functional prognosis after MT remain unclear. Previous studies have speculated that hyperglycemia may directly affect the brain through a variety of mechanisms, for example, it can cause intracellular acidosis in ischemic brain tissue, lead to mitochondrial dysfunction, and aggravate brain damage. In addition, it stimulates the formation of reactive oxygen species and reactive nitrogen species and promotes the development of reperfusion injuries such as cerebral edema and hemorrhagic transformation (10, 25). Several studies have shown that acute hyperglycemia is associated with kidney injury. Data from one of the animal studies showed that glucose injection caused structural and functional changes in glomerular and tubular epithelial cells and induced structural changes in small blood vessels (26). In addition, acute hyperglycemia has adverse effects on the kidney by inducing inflammation, oxidative stress, and apoptosis when stimulated by vascular surgical stress (27). Interestingly, our study demonstrated that postoperative renal function partially mediated the association between AH on functional outcome at 6 months after MT (mediated effect, 25.57%). This suggests that postoperative renal function may play an important role in the relationship between AH and poor outcome of MT patients.

To date, several studies have reported the *association* between postoperative renal dysfunction and functional outcomes. One of these studies reported acute renal dysfunction occurring within 24 to 72 h after MT in AIS patients and was independently associated with increased mortality (28). Covic et al. (29) reported that among 932 AIS patients with or without acute renal dysfunction after MT, the 30-day mortality was 43 and 13%, respectively. Postoperative renal dysfunction also significantly increased the length of hospital stay and hospitalization costs. Consistent with previous findings, our study showed that renal function within 72 h after MT was independently associated with of functional outcome at 6 months. However, the exact mechanism of the relationship between postoperative renal function and long-term functional prognosis of AIS patients remains unclear. One possible explanation is that renal dysfunction may contribute to the exacerbation of neural injury, disrupting the homeostatic interactions between the nervous system and the kidneys. Animal models have shown that acute kidney injury can lead to increased levels of inflammatory proteins KC and G-CSF in brain tissue, inflammatory cells and microglia elevated, the increased expression of GFAP in astrocytes, disruption of blood–brain barrier permeability and behavioral disorders (30). The aggravation of nerve injury is related to higher recurrence rate, mortality and lower quality of life in patients with stroke (31). The decline in postoperative eGFR after MT may be multifactorial, including contrast-induced nephropathy, hemodynamic instability, systemic inflammation, and oxidative stress. Experimental and clinical evidence suggests that acute hyperglycemia may exacerbate renal microvascular dysfunction, oxidative stress, and inflammatory activation, potentially increasing susceptibility to peri-procedural renal injury. In our mediation analysis, admission hyperglycemia was significantly associated with postoperative eGFR reduction, and eGFR partially mediated the relationship between AH and poor functional outcome. Moreover, several studies have suggested that patients with acute renal dysfunction or patients whose kidney function has recovered are more likely to have a recurrent stroke. Wu et al. reported that convalescent patients with acute renal dysfunction have a 1.3-fold higher risk of future stroke recurrence than patients without renal dysfunction (32). Additionally, postoperative renal dysfunction may also reflect postoperative renal dysfunction may reflect various internal homeostasis disturbances in the patient. For example, hemodynamic instability, dehydration caused by inadequate nutritional intake, and physiological disorders, including increased inflammation and oxidative stress, may be associated with the deterioration of stroke prognosis (33). In addition, patients with postoperative renal dysfunction usually have a higher incidence of hospital complications, such as pneumonia, deep venous thrombosis, urinary tract infection, etc., thus aggravating the prognosis of patients (34). However, this study lacks these indicators during hospitalization to verify this explanation.

Our results provide clinically relevant evidence for the treatment of AIS patients, mainly those with AH. Considering that there may be significant differences in clinical outcomes between AH patients and non-AH patients, elevated glucose levels may attenuate the overall benefit of MT treatment. These findings suggest that careful glycemic management, aiming to maintain blood glucose within a safe and reasonable range prior to mechanical thrombectomy (MT), may be beneficial for AIS patients presenting with acute-phase hyperglycemia (AH). In addition, our mediation analysis indicates that postoperative renal function may serve as a potential therapeutic target or monitoring indicator for long-term clinical outcomes in AIS patients with AH.

Despite the prospective design, this study has several limitations to our findings. Firstly, it was a single-center prospective cohort observational cohort study and a multicenter studies are warranted to replicate these results. Secondly, our study lacks some hospitalization data (such as pulmonary infection, deep venous thrombosis, urinary tract infection, etc.) to explore in detail the specific mechanism of the effect of postoperative renal function on prognosis. Patients with missing glucose or creatinine data were excluded, which may introduce selection bias. We did not have longitudinal glucose or eGFR measurements to assess temporal trends. At the same time the regression analyses in this study are intended to explore associations between biomarkers and treatment outcomes. The findings provide important preliminary evidence and candidate indicators for the development and validation of formal clinical prediction models in larger cohorts; however, this study itself does not construct or validate such predictive models. In addition, we lack data on blood glucose and renal function during hospitalization and follow-up, and are unable to explore the relationship between the dynamic changes of blood glucose, renal function and the prognosis of stroke. Detailed information on vascular territory (i.e., anterior versus posterior circulation) and occlusion location was not available in our dataset due to the study design. Given that these factors may influence both treatment response and clinical outcomes, their absence may limit the interpretability of our findings. Future studies incorporating detailed neuroimaging data are warranted to further validate our results. Finally, our adjustment of confounding factors residual confounding cannot be excluded, so the interpretation of this result should be more cautious.

In conclusion, our study suggests that AH is an independently associated with of 6-months functional outcome after MT in AIS patients with or without diabetes, and this association may be partially mediated in part by postoperative renal function.

## Data Availability

The original contributions presented in the study are included in the article/supplementary material, further inquiries can be directed to the corresponding authors.
